# Supplementary material for: SARS-CoV-2 Omicron neutralization by therapeutic antibodies, convalescent sera, and post-mRNA vaccine booster
Source: bioRxiv. 2021 Dec 28:2021.12.22.473880. Preprint. [Version 2] doi: 10.1101/2021.12.22.473880 (PMC8722594; doi:10.1101/2021.12.22.473880)
Supplement: Supplement 1 — Table S1: SARS-CoV-2 genotypes for the infecting variants [file media-1.pdf]

Supplementary Table. SARS-CoV-2 genotypes for the infecting variants.

Sample #	Pangolin 3.1.17 (2021-12-06)	Spike Substitutions	Spike Deletions
Conv-01	AY.74	S:T19R,S:G142D,S:R158G,S:A222V,S:L452R,S:T478K,S:D614G,S:P681R,S:D950N	S:E156-,S:F157-
Conv-02	AY.25	S:T19R,S:S112L,S:G142D,S:R158G,S:L452R,S:T478K,S:D614G,S:P681R,S:D950N	S:E156-,S:F157-
Conv-03	AY.47	S:T19R,S:G142D,S:R158G,S:A222V,S:V289I,S:L452R,S:T478K,S:D614G,S:P681R,S:D950N	S:E156-,S:F157-
Conv-04	B.1.617.2	S:T19R,S:K77T,S:G142D,S:R158G,S:G181V,S:L452R,S:T478K,S:D614G,S:A653V,S:P681R,S:D950N	S:E156-,S:F157-
Conv-05	B.1.617.2	S:T19R,S:K77T,S:G142D,S:R158G,S:G181V,S:L452R,S:T478K,S:D614G,S:A653V,S:P681R,S:D950N	S:E156-,S:F157-
Conv-06	AY.14	S:T19R,S:G142D,S:R158G,S:L452R,S:T478K,S:D614G,S:P681R,S:D950N	S:E156-,S:F157-
Conv-07	AY.14	S:T19R,S:G142D,S:R158G,S:L452R,S:T478K,S:D614G,S:P681R,S:D950N	S:E156-,S:F157-
Conv-08	AY.25	S:T19R,S:S112L,S:G142D,S:R158G,S:L452R,S:T478K,S:D614G,S:P681R,S:D950N	S:E156-,S:F157-
Conv-09	B.1.617.2	S:T19R,S:T95I,S:G142D,S:R158G,S:L452R,S:T478K,S:D614G,S:P681R,S:D950N,S:G1124V	S:E156-,S:F157-
Conv-10	AY.62	S:T19R,S:G142D,S:R158G,S:A222V,S:L452R,S:T478K,S:D614G,S:P681R,S:G946V,S:D950N	S:E156-,S:F157-
Conv-11	AY.25	S:T19R,S:G142D,S:R158G,S:L452R,S:T478K,S:D614G,S:P681R,S:D950N	S:E156-,S:F157-
Conv-12	AY.44	S:T19R,S:T22I,S:G142D,S:R158G,S:L452R,S:T478K,S:D614G,S:P681R,S:D950N	S:E156-,S:F157-
Conv-13	AY.119	S:T19R,S:T95I,S:G142D,S:R158G,S:L452R,S:T478K,S:D614G,S:P681R,S:D950N	S:E156-,S:F157-
Conv-14	B.1.617.2	S:T19R,S:G142D,S:R158G,S:L452R,S:T478K,S:D614G,S:P681R,S:D950N	S:E156-,S:F157-
Conv-15	B.1.617.2	S:T19R,S:G142D,S:R158G,S:L452R,S:T478K,S:D614G,S:P681R,S:D950N,S:V1264L	S:E156-,S:F157-
Conv-16	B.1.617.2	S:T19R,S:T95I,S:G142D,S:R158G,S:L452R,S:T478K,S:D614G,S:P681R,S:D950N	S:E156-,S:F157-
Conv-17	B.1.617.2	S:T19R,S:G142D,S:M153I,S:R158G,S:A222V,S:L452R,S:T478K,S:D614G,S:P681R,S:D950N	S:E156-,S:F157-
Conv-18*	B.1.351		
Conv-19	B.1.351	S:D80A,S:D215G,S:K417N,S:E484K,S:N501Y,S:D614G,S:A701V	
Conv-20	B.1	S:D614G	
Conv-21	B.1	S:D614G	
Conv-22	B.1	S:D614G	
Conv-23	B.1	S:D614G	
Conv-24	B.1	S:D614G	
Conv-25	B.1	S:D614G	
Conv-26	B.1	S:D614G	
Conv-27	B.1	S:D614G	
Conv-28	B.1	S:D614G	
Conv-29	B.1	S:D614G	
Conv-30	B.1.1.7	S:N501Y,S:A570D,S:D614G,S:P681H,S:T716I,S:S982A,S:D1118H	S:H69-,S:V70-,S:Y144-
Conv-31	B.1.1.7	S:N501Y,S:A570D,S:D614G,S:P681H,S:T716I,S:S982A,S:D1118H	S:H69-,S:V70-,S:Y144-
Conv-32	B.1.1.7	S:N501Y,S:A570D,S:D614G,S:P681H,S:T716I,S:S982A,S:D1118H,S:K1191N	S:H69-,S:V70-,S:Y144-
Conv-33	B.1.1.7	S:N501Y,S:A570D,S:D614G,S:P681H,S:T716I,S:S982A,S:D1118H	S:H69-,S:V70-,S:Y144-
Conv-34	B.1.1.7	S:N501Y,S:A570D,S:D614G,S:P681H,S:T716I,S:S982A,S:D1118H	S:H69-,S:V70-,S:Y144-
Conv-35	B.1.2	S:D614G	
Conv-36	B.1.2	S:G257D,S:D614G	
Conv-37	B.1.2	S:D614G	
Conv-38	B.1.2	S:D614G	
Conv-39	B.1.2	S:D614G	
Conv-40	B.1.2	S:D614G	

*The sequencing information from this individual was not avalable (see methods)
